# Evaluation of a probe hybridization quantitative polymerase chain reaction assay for *Cryptosporidium serpentis* in eastern indigo snakes (*Drymarchon couperi*)

**DOI:** 10.1007/s00436-022-07676-4

**Published:** 2022-09-29

**Authors:** James E. Bogan, James F. X. Wellehan, Michael M. Garner, April L. Childress, Bethany Jackson

**Affiliations:** 1 School of Veterinary Medicine, Murdoch University, 90 South Street, Murdoch, WA 6150 Australia; 2 Central Florida Zoo’s Orianne Center for Indigo Conservation, 30931 Brantley Branch Road, Eustis, FL 32736 USA; 3 Department of Comparative, Diagnostic, and Population Medicine, College of Veterinary Medicine, University of Florida, Gainesville, FL 32610 USA; 4 Northwest ZooPath, 654 West Main Street, Monroe, WA 98272 USA

**Keywords:** Cryptosporidiosis, Indigo snake, qPCR, Sensitivity, Specificity, Reptile

## Abstract

A probe-hybridization quantitative polymerase chain reaction assay specific for *Cryptosporidium serpentis* (qPCR) has been developed and shown to be extremely sensitive in the laboratory, but clinical sensitivity and specificity for this test are lacking. To approximate the sensitivity and specificity of the *C. serpentis* qPCR, the medical records from a captive snake colony were reviewed, and between November 2015 and June 2021, 63 eastern indigo snakes (*Drymarchon couperi*) were necropsied. Of these 63 snakes, 11 had qPCR performed on gastric biopsies collected at the time of necropsy, 8 had qPCR performed on samples collected by gastric swab within 35 days of necropsy, and 34 had qPCR performed on samples collected by cloacal swab within 84 days of necropsy. The qPCR results were then compared to the post-mortem histological findings, where all three sampling techniques had a 100% specificity. The sensitivity was highest in samples collected at necropsy (100%, CI: 63.06 – 100%) followed by the ante-mortem testing: gastric swab (87.50%, CI: 42.13 – 99.64%) and cloacal swab (66.67%, CI: 44.68 – 84.37%).

## Introduction

*Cryptosporidium serpentis* is a gastrotropic apicomplexan parasite that infects diverse species of squamates and is spread through a fecal–oral route with intermittent oocyst shedding (Karasawa et al. 2002). Infected snakes typically develop severe gastric mucosal hypertrophy which occludes the gastric lumen, leading to anorexia, regurgitation, vomiting, and weight loss (Cranfield and Graczyk 2006). These changes prevent proper digestion and nutrient uptake, and snakes typically die of starvation and/or sepsis.

A presumptive diagnosis of squamate gastric cryptosporidiosis (SGC) can be achieved through any combination of clinical signs, cytologic findings, and molecular analyses. The diagnosis of *C. serpentis* infection is more challenging for snakes that do not have typical clinical signs. Sampling techniques include cloacal swab, cloacal lavage, gastric swab, gastric lavage, and/or gastric biopsy, with limitations related to sampling ease and diagnostic accuracy (Bogan 2019). Samples from the stomach have been shown to be superior to samples from the cloaca, with the diagnostic yield highest when the sample is collected 3 days following a meal (Graczyk et al. 1996). When histologic evaluation of a gastroscopic biopsy was compared to conventional PCR and sequencing of gastric lavage for diagnosing occult SGC, both sampling techniques had 100% specificity (Cerveny et al. 2012). However, the histologic evaluation of a gastroscopic biopsy was reported to have a 71.4% sensitivity compared to a 20% sensitivity for conventional PCR and sequencing of a gastric lavage (Cerveny et al. 2012).

Probe hybridization quantitative PCR (qPCR, real-time PCR) is a technique that uses hybridization of a sequence-specific probe during the PCR reaction to validate product identity. As the polymerase advances past the hybridized probe, exonuclease activity digests the probe and labeled dyes are released and spectrophotometrically measured. Specific product synthesis can therefore be determined as the reaction progresses, providing not only qualitative information regarding presence, but also quantitative information. This technique is substantially less labor and resource intensive than product identification by sequencing and is often found to be more sensitive than standard PCR with sequencing (Vidal et al. 2011).

The objective of this study was to determine the clinical sensitivity and specificity of a probe-hybridization quantitative polymerase chain reaction assay specific to *C. serpentis* when used on cloacal or gastric swabs collected from snakes.

## Methods

### qPCR development

DNA was extracted from all samples (DNeasy Blood and Tissue kit; Qiagen, Germantown, MD), and DNA concentration was measured by spectrophotometry and double checked by comparison to standards on electrophoresis (NanoDrop 1000 spectrophotometer, Thermo Fisher, Gaithersburg, MD; low mass DNA ladder, Thermo Fisher). All extracts were aliquoted and stored at − 80 °C.

A probe hybridization quantitative polymerase chain reaction assay (qPCR) specific to *C. serpentis* was developed. For the standard curve, nested consensus primers were designed for the *Cryptosporidium *actin gene by comparison of homologous *Cryptosporidium* sp. sequences available in GenBank and tested in silico for secondary structure concerns and mispriming using Primer3 prior to use (Untergasser et al. 2012). Primers CryptActFouter (5′-CGTGARAGAATGACYCARATTATGTT-3′) and CryptActRouter: (5′-CWGGATACATWGTWGTACCACCAGA-3′) were used for the first round and CryptActFinner (5′-ATACHGTHCCWATTTATGARGGWTATGC-3′) and CryptActRinner (5′-TCCTTACGRATATCRAGRTCACA-3′) for the second round. The PCR product was resolved in a 1% agarose gel, the band was excised, and DNA was extracted (QIA- quick gel extraction kit; Qiagen) and sequenced by Sanger methodology to confirm identity. A spectrophotometer (NanoDrop, Thermo Fisher Scientific Inc., Waltham, MA) was used to measure the concentration, and copy numbers were calculated. The sample was diluted to 10^6^ copies per 7 μL, and sets of tenfold serial dilutions were then made from 10^6^ down to 10 copies per 7 μL. Control standards were run in duplicate wells at each concentration as a standard curve in each run, with an additional two negative control wells.

Primers and probes specifically targeting the *C serpentis* actin gene were designed for specific amplification and detection of *C. serpentis* by comparison to other homologous *Cryptosporidium* sp. sequences available in GenBank and tested in silico for secondary structure concerns and mispriming using Primer3 prior to use (Untergasser et al. 2012). Primers were SerpentisF (5′-GCTGGTCGTGATTTAACTGATT-3′), SerpentisR (5′-TTTCTGCAGTTGTTGTAAAGCTG-3′), and SerpentisProbe (FAM-TTGATGAAAATTTTGCATGATCGTGGT-BHQ1). All qPCR reactions took place on a 96-well reaction plate (MicroAmp Fast optical reaction plates; Applied Biosystems). Each reaction well consisted of a 20-μL solution containing 1 μL each of forward primer, reverse primer, and probe, 7 μL of sample, and 10 μL of TaqMan master mix (TaqMan Fast Universal PCR Master Mix 2X; Applied Biosystems). Each reaction was run in duplicate. A eukaryotic 18S rRNA endogenous control kit (VIC/MGB Probe; Applied Biosystems) was used to confirm the presence of amplifiable DNA in each sample. Nuclease-free water is used in 6 wells to serve as non-template controls. The reactions were amplified (7500 Fast Real-Time PCR System; Applied Biosystems). Cycling conditions included initial denaturation at 95 °C for 20 s, then 40 cycles of 95 °C for 3 s and 60 °C for 30 s. The function of the qPCR probes and primers was assessed by evaluating *R*^2^ values and slopes of the standard curves, calculated using the software included with the ABI 7500 fast equipment. Twenty sequence-confirmed *C. serpentis*-positive samples, as well as samples positive for *Cryptosporidium avium*, *Cryptosporidium varanii*, *Cryptosporidium muris*, *Cryptosporidium tyzzeri,* and the unnamed species first reported from Japanese grass snakes, were run to examine assay specificity.

The standard curve of the actin probe hybridization qPCR assay had a slope of − 3.785, indicating 83.7% efficiency, and *R*^2^ of 0.997. The assay successfully amplified all dilutions from 10 to 1,000,000 control copies. All control *C. serpentis*-positive samples were positive by qPCR, and all *C. avium*, *C. varanii*, *C. muris*, *C. tyzzeri*, and unnamed Japanese grass snake species positive samples were negative by qPCR. The qPCR assay used here was capable of detecting at least as few as 10 copies, but this does not take losses during extraction into account.

### Prevalence of *C.serpentis* in a captive snake colony

From November 2015 through June 2021, 381 eastern indigo snakes (*Drymarchon couperi*) housed in a conservation breeding colony (Central Florida Zoo’s Orianne Center for Indigo Conservation (OCIC), Eustis, FL, USA) were screened for the presence of *C. serpentis*. Of these, 64/381 (16.8%) *D. couperi* tested positive for *C. serpentis* through molecular analyses.

### Sampling sites

Medical records for all snakes housed at OCIC from November 2015 through June 2021 were reviewed. Sixty-three snakes had died and were necropsied within that period. Cause of death for these 63 snakes included SGC (28), trauma (8), septicemia (7), yolk coelomitis (6), hyperviscosity-like syndrome (5), cardiomyopathy (4), renal failure (2), reproductive neoplasia (2), and biliary hyperplasia (1). The 28 EIS that had SGC as cause of death had varying degrees of clinical signs including anorexia, regurgitation, and weight loss. None of these EIS had visible mid-body swellings. Of these 63 snakes, 11 had *C. serpentis* qPCR performed on gastric tissue at the time of necropsy, 8 had *C. serpentis* qPCR performed on samples collected by gastric swab within 35 days of necropsy, and 34 had *C. serpentis* qPCR performed on samples collected by cloacal swab within 84 days of necropsy. In addition to the necropsied snakes, 70 other snakes within this collection had gastroscopic biopsies for histologic examination and concurrent *C. serpentis* qPCR ante-mortem.

Of the 70 individuals that had gastroscopic biopsies, each had 15–20 biopsies collected from the pyloric, fundic, and antral regions of the stomach. Biopsies were obtained by passing a 2-mm endoscopic biopsy forceps through the working channel of a video endoscope (D-VS-8015, MDS-Vet, Valrico, FL, USA) after the endoscope was advanced into the stomach and direct visualization of biopsy target sites was made. Half of the collected biopsies were frozen on wet ice and submitted for qPCR testing for *C. serpentis*, and half placed in 10% neutral buffered formalin for histological evaluation. Samples for histologic evaluation were processed routinely, sectioned at 5 μm, mounted on frosted glass slides, stained with hematoxylin and eosin, and examined by one board-certified veterinary pathologist (MMG). Disease status was determined by histologic examination of gastroscopically obtained biopsies, whereby a positive case was based on visual identification of cryptosporidia within the gastric mucosa and associated lesions. A negative histologic examination was based on the presence of normal gastric mucosa and absence of cryptosporidia.

### Statistical analysis

Agreement between tests was calculated using Cohen’s Kappa as a formula in Microsoft Excel (version 2108, Microsoft Corporation, Redmond, WA, USA). Sensitivity, specificity, positive predictive value, negative predictive value, and accuracy were calculated with statistical software (MedCalc Software Ltd. Diagnostic test evaluation calculator. https://www.medcalc.org/calc/diagnostic_test.php (Version 20.009; accessed July 14, 2021)), using 16.8% as the prior (assumed) prevalence in this population.

## Results and discussion

Cohen’s Kappa analysis of the results demonstrated excellent agreement (*k* = 1.000) between histologic and qPCR analysis of gastric samples collected during necropsy (Table 1). There was moderate agreement between qPCR analysis of samples collected by gastroscopic biopsy and histologic analysis of gastroscopic biopsy where cryptosporidia were visualized (*k* = 0.588). There was also moderate agreement between qPCR analysis of samples collected by gastric or cloacal swabs when compared to histologic evaluation of gastric samples collected either by gastroscopy or at necropsy within three months (*k* = 0.600 and 0.541, respectively). There was fair agreement (*k* = 0.494) between qPCR analysis of samples collected by gastroscopic biopsy and histologic analysis of gastroscopic biopsy when lesions consistent with cryptosporidiosis were present, regardless of whether cryptosporidia were visualized (Table 2). Using histologic results obtained at necropsy as the “Gold Standard,” qPCR analysis of gastric biopsies obtained during necropsy was in complete agreement and had 100% sensitivity and 100% specificity. Likewise, qPCR analyses of both stomach swabs and cloacal swabs collected antemortem had 100% specificity. Of the antemortem sampling techniques, however, qPCR on a stomach swab collected 3 days after a meal was more sensitive than a qPCR on a cloacal swab (Table 1).

**Table 1:** Comparison of various sampling methods for diagnosing squamate gastric cryptosporidiosis in eastern indigo snakes (*Drymarchon couperi*)

	*n*	*κ*	Sensitivity	Specificity	PPV	NPV
			(95% CI)	(95% CI)	(95% CI)	(95% CI)
Gastric Bx	11	1.000	100%	100%	100%	100%
			(63.06–100.00%)	(29.24–100.00%)		
Gastric swab	8	0.600	87.50%	100%	100%	97.54%
			(42.13–99.64%)	(2.50–100%)		(84.96–99.53%)
Cloacal swab	34	0.541	65.67%	100%	100%	93.69%
			(44.68–84.37%)	(69.15–100%)		(89.40–96.32%)

Gastric Bx = comparison of *C. serpentis* qPCR on gastric tissue collected at necropsy versus histologic analysis of gastric tissue collected at necropsy; Gastric Swab = comparison of *C. serpentis* qPCR on gastric swab collected within 35 days of gastric histologic evaluation (necropsy or gastroscopic biopsy); Cloacal Swab = comparison of *C. serpentis* qPCR on cloacal swab collected within 84 days of gastric histologic evaluation (necropsy or gastroscopic biopsy); *n* = number of snakes sampled; *κ* = Cohen’s kappa coefficient; 95% CI = 95% confidence interval. Calculations were made based on the prevalence of 16.8% within the collection. Guidelines for interpreting *κ* values: very good agreement, 0.81–1.00; good agreement, 0.61–0.80; moderate agreement, 0.41–0.60; fair agreement, 0.21–0.40; and poor agreement, less than 0.20

**Table 2:** Comparison of gastroscopic sampling methods for diagnosing squamate gastric cryptosporidiosis in eastern indigo snakes (*Drymarchon couperi*)

	*n*	*κ*	Sensitivity	Specificity	PPV	NPV
			(95% CI)	(95% CI)	(95% CI)	(95% CI)
Organism and lesion	70	0.588	100.00%	62.50%	35.00%	100%
			(88.43–100.00%)	(45.80–77.27%)	(26.52–44.55%)	
Lesions only	70	0.340	75.81%	87.50%	55.05%	94.71%
			(63.26–85.78%)	(47.35–99.68%)	(16.30–88.51%)	(91.47–96.76%)

Organism and lesion = comparison of *C. serpentis* qPCR on gastric tissue collected by gastroscopy versus histologic analysis of gastric tissue collected by gastroscopy where cryptosporidial organisms were visualized; Lesions only = comparison of *C. serpentis* qPCR on gastric tissue collected by gastroscopy versus histologic analysis of gastric tissue collected by gastroscopy where pathologic changes consistent with cryptosporidiosis were present regardless if cryptosporidial organisms were seen; *n* = number of snakes sampled; *κ* = Cohen's kappa coefficient; 95% CI = 95% confidence interval. Calculations were made based on the prevalence of 16.8% within the collection. Guidelines for interpreting *κ* values: very good agreement, 0.81–1.00; good agreement, 0.61–0.80; moderate agreement, 0.41–0.60; fair agreement, 0.21–0.40; and poor agreement, less than 0.20

A previous real-time PCR methodology for the *hsp70* gene of *C. serpentis* has been reported (da Silva et al. 2014). Their assay had good concordance with a nested PCR/sequencing assay, with a kappa index of 0.871; however, it was not compared with histologic results. Their assay also differed from ours in that it utilized dye incorporation rather than probe hybridization, with specificity depending on melt curves rather than probe binding.

Analysis of our *C. serpentis* qPCR demonstrates that the specificity is 100% for samples collected during necropsy and for ante-mortem samples collected either by gastric or cloacal swabs. This agrees with the screening done prior to analyzing snake samples, where DNA extracts of clinical samples that contained multiple other *Cryptosporidium* species were used. The specificity of the *C. serpentis* qPCR on gastroscopically collected biopsies was lower, depending on the definition of a positive case. If a positive case is defined by only seeing the organism, then the specificity of the qPCR is 62.50% (CI: 45.80 – 77.27%). If the definition is expanded to include samples with lesions consistent with SGC even if the organism was not seen, then the specificity of the qPCR increases to 87.50% (CI: 47.35 – 99.68%). This may indicate that the qPCR is actually more specific than histologic exam when small tissue samples are analyzed, since a 2-mm pinch biopsy may identify the lesion but not identify the organism in the lesion. Additionally, since qPCR was not performed on the same samples evaluated histologically, it is possible that the samples containing observable cryptosporidia were only sent for molecular analysis. Since any formalin fixation can reasonably be expected to markedly decrease sensitivity of nucleic acid-based tests, direct comparison on the corresponding paraffin embedded biopsy material is not recommended. Increasing sampling size by processing multiple samples from individual animals may result in more leptokurtic results.

The sensitivity is also dependent upon the sampling technique used. Tissue biopsy for qPCR analysis has the highest sensitivity (100%), for necropsy and gastroscopy. A gastric swab collected 3 days after a meal had higher sensitivity (87.50%, CI: 42.13 – 99.64%) when compared to cloacal swab (66.67%, CI: 44.68 – 84.37%). Repeating multiple ante-mortem tests in series may increase the sensitivity, with the sensitivity for repeated tests estimated using the formula: Sens_*calc*_ = 1 − (1 − Sens)^*n*^. So, in theory, the sensitivity of stomach swabs could be increased from 87.50 to 99.80% and cloacal swabs from 66.67 to 96.30% by repeating the swab three times (0.9980 = 1 − (1 − 0.8750)^3^ and 0.9630 = 1 − (1 − 0.6667)^3^, respectively).

The authors acknowledge that latent class analysis is increasingly being used to further evaluate sensitivity and specificity where disease status of individuals cannot be definitively confirmed (e.g., where no necropsy is performed) (MacLean and Dendukuri 2020). Future research could use this approach to investigate broader data sets and derive latent class models which may estimate these parameters with greater precision.

Since the prepatent period of *C. serpentis* infection has been determined to be between 12 and 28 weeks (84 to 196 days) in *Pantherophis* spp. (Cranfield and Graczyk 1994), comparing the sample collection within 84 days from necropsy results should be appropriate. If the prepatent period was shorter than 84 days, then a false negative result may have occurred in the ante-mortem diagnostic test. Euthanizing the animal after collecting the sample and comparing those results with the necropsy results would remove the prepatent factor. Since these animals are part of a captive breeding colony of an endangered species, this method of analysis is impractical.

The authors also acknowledge the limitations of retrospective analysis of data (Talari and Goyal 2020). Effort was made to limit confounders by limiting the study to EIS with antemortem testing performed within the known prepatent period timeframe prior to necropsy. Care must be taken not to over generalize results of retrospective analysis. The sensitivity of this assay for given sampling sites in non-EIS squamates may differ from that found in EIS.

## Conclusion

These results demonstrate when screening for *C. serpentis*, qPCR analysis of a gastric swab collected three days after a meal is more sensitive than qPCR analysis of a cloacal swab. To increase the likelihood of detecting occult SGC when screening snakes, collecting multiple samples is warranted, even when the most sensitive testing methods such as qPCR are used. If repeated testing is not available, the practitioner must determine whether the sensitivity a single test result is sufficient for their current disease screening needs. Based on the data provided within this study, a minimum of three qPCR tests should be performed on gastric swabs to increase the likelihood of detecting *C. serpentis* in occult infections to a predicted 99.8%, and to ensure that biosecurity and management decisions are based on the best available evidence.

## Data Availability

Not applicable.
